# Autologous platelet-rich plasma separation technology used in the same patient receiving 2 complicated aortic surgeries within a short time period: A case report

**DOI:** 10.1097/MD.0000000000017415

**Published:** 2019-10-04

**Authors:** Yi Chang, Rongwei Zhang, Ayong Tian

**Affiliations:** a Department of Anesthesiology; b Department of Gerontology and Geriatrics, the first Affiliated Hospital of China Medical University, Shenyang, China.

**Keywords:** aortic surgery, autologous platelet-rich plasma, case report, coagulation

## Abstract

**Rationale:**

Autologous platelet-rich plasma (PRP) separation technology has been widely used in various clinical therapies, and has achieved good results, especially in aortic surgeries.

**Patient concerns:**

A 50-year-old man who was diagnosed with aortic dissection (Stanford B type), a thoracoabdominal aortic aneurysm, and grade 2 hypertension underwent 2 complicated aortic surgeries within 4 months.

**Diagnoses:**

aortic dissection (Stanford B type).

**Interventions:**

PRP separation used as a blood protection measure was employed in both 2 surgeries.

**Outcomes:**

The patient's coagulation function recovered well after the surgeries. The amount of allogeneic blood products used in the perioperation was small.

**Lessons:**

PRP separation technology combined with blood salvage and warming of blood and fluid transfusion in the aortic surgery has been proved to be feasible and beneficial.

## 1 Introduction

**Figure 1:**
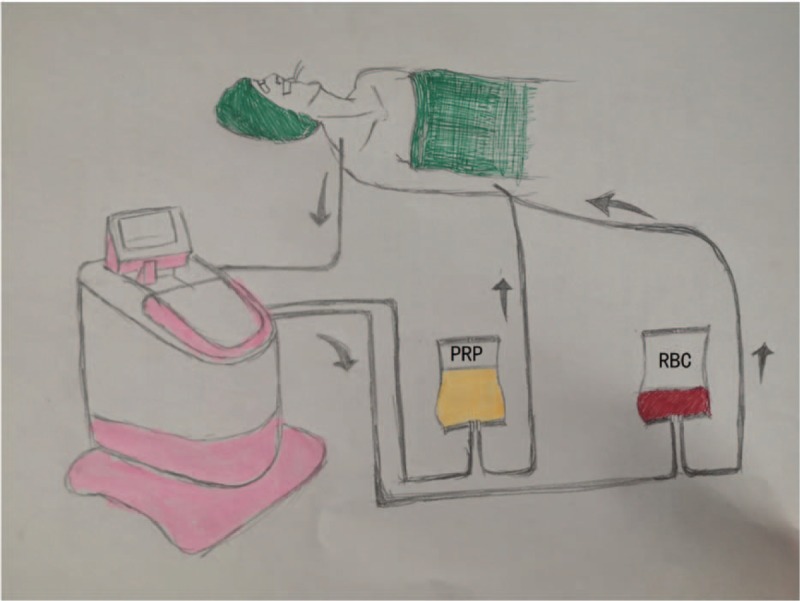
Autologous blood was collected from the right internal jugular vein and separated using an Autotransfusion Blood Separator device before heparinize.

Coagulation is an important complication of aortic dissection aneurysm surgery due to severe surgical trauma, prolonged duration of cardiopulmonary bypass (CPB), and the need for deep hypothermic circulatory arrest. In addition, many patients may require a second thoracotomy to stop the bleeding due to a large postoperative drainage volume. To achieve full use of the innate coagulation system, autologous platelet-rich plasma (PRP) separation technology has been employed in various cardioaortic centers throughout the world, with Professor Zhou from the Texas Medical Center as a pioneer of this research^[1,2]^ (Fig. 1). Autologous PRP separation technique refers to the collection of autologous blood that is separated into PRP and red blood cells (RBCs) fractions after anesthesia and prior to heparinization, which was neutralized by protamine administration and infusion back to the patient after termination of CPB. The main advantage of this technology is the ability to retain part of the autologous platelets and plasma to avoid the activation and consumption of CPB, and to facilitate postoperative recovery of patients via the autologous-coagulation system. This article briefly describes a patient who underwent 2 complicated aortic surgeries with plasma separation within a short period of time. The patient's coagulation function recovered well after the surgeries.

## 2 Case presentation

The patient was a 50-year-old man with a body mass index of 25.7 kg/m^2^ and a 1-year history of hypertension who was preoperatively diagnosed with aortic dissection (Stanford B type), a thoracoabdominal aortic aneurysm, and grade 2 hypertension. The treatment plan consisted of staged reconstructive surgeries of the ascending aorta, aortic arch, and full thoracic and abdominal aorta. The patient has provided informed consent for publication of the case.

### 2.1 Anesthesia and surgical procedures

As the first stage of the surgery, the total aortic arch was replaced, and a stent was placed in the descending aorta. With routine monitoring, the patient was induced of intravenous anesthesia and maintenanced of intravenous-inhalational combined anesthesia. Intermittent blood sampling was conducted for blood gas and electrolyte analysis. Thromboelastography (TEG) was employed to monitor the patient's coagulation function. During surgery, external warming and warming of transfused blood were employed to prevent hypothermia. Autologous blood was collected through the right internal jugular vein and separated into 878 mL of PRP and 497 mL of RBCs using an Autotransfusion Blood Separator device (Xtra Sorin Group Deutschland GmbH). The PRP was stored under continuous shaking at room temperature (22–24 °C). The Autotransfusion Blood Separator device was used to achieve and concentrate the whole blood lost during the time from cutting the skin to closing the chest.

While the right axillary artery and the right femoral artery were catheterized respectively, a median incision was made and both superior and inferior vena cava were incubated to establish a cardiopulmonary bypass. Selective cerebral perfusion was performed to maintain regional cerebral oxygen saturation (rScO_2_) at least 55%. A 28 × 80-mm stent was placed in the descending aorta. Then a 28-mm-diameter 4 branches aortic graft was used for replacement, the proximal end of which was anastomosed with the end of the autologous vessel and the distal end of which was anastomosed with the proximal end of the descending aortic stent. Circulation arrest lasted for a total of 22 minutes. The ascending aorta was blocked for a total of 92 minutes, with the cardiopulmonary bypass stopped after 22 minutes.

After heparin neutralization with protamine, PRP and autologous RBCs were transfused back to the patient. A total of 251 mL of concentrated RBCs were collected during surgery. Another 2 U of allogeneic RBCs, 2 U of platelets, and 20 U of cryoprecipitate were administered. The total urine volume was 750 mL. The surgery lasted for 4.5 hours and the patient was admitted to the cardiac surgery intensive care unit (CS-ICU) after surgery with endotracheal intubation.

### 2.2 Postoperative condition

After the first surgery, the patient receives no allogeneic blood products. The platelet count before, immediately after, and 24 hours after the surgery were 218 × 10^9^/L, 173 × 10^9^/L, and 198 × 10^9^/L, respectively. The MA value of TEG before, immediately after, and 24 hours after the surgery were 71.8, 69.8, and 70.1 mm, respectively. The ventilator was withdrawn at 18 hours after surgery and the patient was discharged from the CS-ICU on postoperative day (POD) 4 and discharged on POD 35. At discharge, the general condition of patient was good, with normal limb activity and no nervous system-related complications.

### 2.3 The second surgery procedures

Four months later, as the second stage of the surgery, thoracoabdominal aortic replacement was performed. The patient's body mass index was 24.7 kg/m^2^ and thoracoabdominal computed tomography angiography suggested that after surgery of the aortic arch and descending aorta, an aneurysm with thrombosis had formed in the thoracoabdominal aorta, as determined by a low-density linear shadow in the distal segment of the aortic arch to the distal part of the abdominal aorta. The lumen was divided into true and false chambers.

On the day before surgery, the L3–4 subarachnoid space was punctured to insert the drainage tube, which was connected with the cerebrospinal fluid drain and pressure monitoring system, and properly fixed after ensuring smooth drainage and normal pressure. Conventional intravenous anesthesia was induced after entering the operating room and an endobronchial blocker tube was placed through the endotracheal tube. The position of the endobronchial blocker tube was confirmed by the fiberoptic bronchoscope. Intermittent drainage of cerebrospinal fluid (10 mL/h) was performed to maintain cerebrospinal fluid pressure <10 mmHg whether intra or post-operation. Blood and electrolytes were analyzed intermittently, and blood coagulation function was monitored by TEG. During surgery, external warming and warming of the transfused blood was employed to prevent hypothermia.

Autologous blood separation and recovery were used for blood preservation. Autologous blood was collected from the right internal jugular vein and separated using an Autotransfusion Blood Separator device. A total of 865 mL of PRP and 521 mL of RBCs were isolated. The device was then used to achieve and concentrate the whole blood lost during the time from cutting the skin to closing the chest.

Surgery was performed with the patient in the right lateral position, with the upper body facing 90° to the right and the lower body at 45°. A left thoracic posterolateral side and left abdominal lateral side combined incision was made, with entry into the chest made at the fourth intercostal space. After systemic heparinization, arterial intubation was performed before the distal iliac artery branch of the abdominal aorta, after that an intravenous catheter was placed to the right atrium entrance through the left femoral vein. The proximal aorta was blocked at the distant end of the aortic arch with the distal thoracic aorta blocked at the T3 level for partial extracorporeal circulation. The main blood vessels and thoracoabdominal aorta were replaced with 26-mm-diameter bifurcated artificial vessels.

After heparin neutralization with protamine, PRP and autologous RBCs were transfused back to the patient. A total of 1353 mL of concentrated RBCs (hematocrit 49%) were collected during surgery. Another 4 U of allogeneic concentrated RBCs, 4 U of platelets, 20 U of cryoprecipitate, and 4000 mL of crystalloid solution were administered. The total urine volume was 900 and 70 mL of cerebrospinal fluid was drained. The surgery lasted for 8 hours and after removal of the endobronchial blocker tube, the patient was admitted to the CS-ICU with tracheal intubation.

### 2.4 Postoperative condition after the second surgery

The patient received only 400 mL of allogeneic plasma after surgery. Cumulatively, 1019 mL of cerebrospinal fluid was drained during the perioperative period. There were no significant differences in platelet counts on the first morning after and 24 hours after surgery, as compared with before surgery (232 × 10^9^/L and 175 × 10^9^/L vs 191 × 10^9^/L, respectively). The MA value of TEG before, immediately after, and 24 hours after the surgery were 66.7, 64.3, and 68.8 mm, respectively. The ventilator was withdrawn at 68 hours after surgery and the patient was discharged from the CS-ICU on POD 8 and discharged on POD 23. At discharge, the general condition of patient was good with normal limb activity and no nervous system-related complication (Tables 1–3).

**Table 1:**
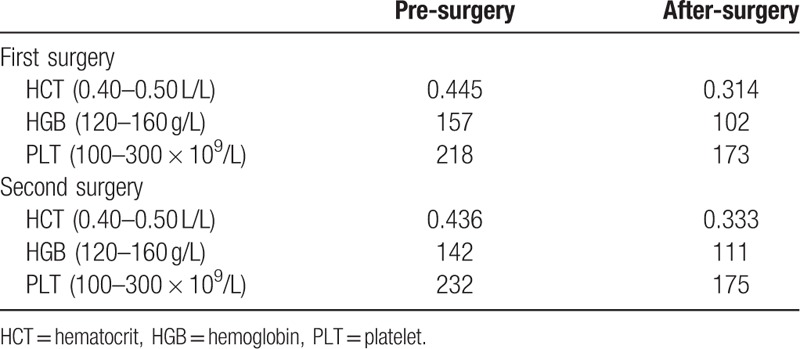
The salient lab parameters.

**Table 2:**
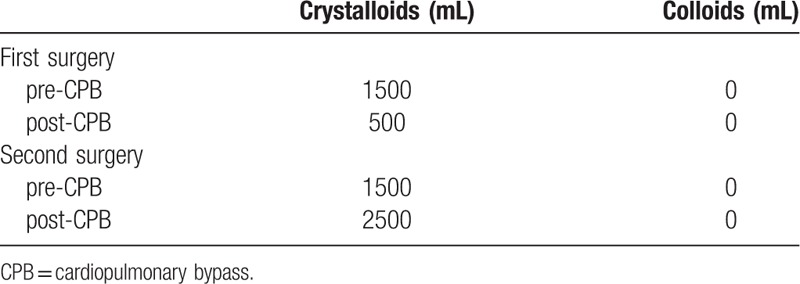
Volume of crystalloids and colloids.

**Table 3:**
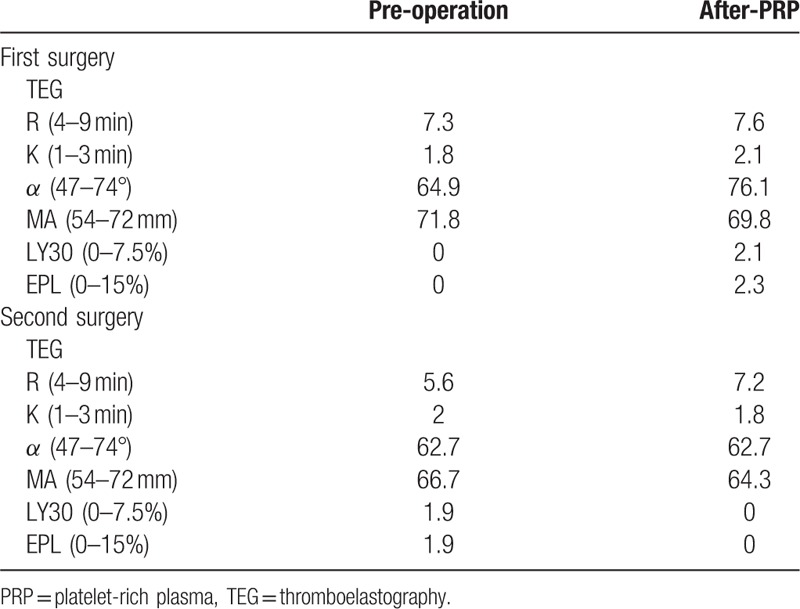
TEG of 2 surgeries.

## 3 Discussion

Perioperative anesthesia management is rather complicated with a prolonged surgical duration and complicated procedure with rapidly changing conditions of the patient. Such care covers almost every aspect of anesthesia management: protection of almost all vital organs (i.e., brain, spinal cord, heart, and kidneys),^[3,4]^ but also focused on blood protection, especially protection and functional recovery of the coagulation system.^[5]^

Blood protection and coagulation recovery are very important in aortic surgery, especially blood separation during the procedure. Mean transfusion rate of packed red blood cells was reduced by 34%, fresh frozen plasma by 52.8%, cryoprecipitate by 70%, and platelets by 56.7% in the aPRP group (*P* < .02). Hospital length of stay (9.4 ± 5.3 days vs 12.7 ± 6.3 days; *P* < .014) and transfusion costs ($1396 ± $1755 vs $2762 ± $2267; *P* < .004) were reduced in the aPRP group.^[1,2]^ In fact, platelet and plasma apheresis have long been used in other clinical areas. In recent years, with the rapid development of diagnostic and therapeutic technologies, periprocedural autologous blood separation, recovery, and transfusion technologies have also been rapidly implemented.^[6]^ The role of autologous platelets and plasma in coagulation is extremely important, especially to preserve platelet function. As long as >20% of autologous platelets are preserved, normal platelet aggregation function can be basically maintained. The widespread use of TEG in recent years has provided clinical anesthesiologists with a more targeted tool for coagulation recovery.^[7]^

In this case, the patient underwent 2 major surgical aortic surgeries with a period of 4 months. It is relatively rare for the same individual to require 2 major aortic surgeries within such a short period of time, but this case fully reflects the advantages of autologous blood separation. Blood separation was employed in both surgeries to collect a sufficient amount of PRP, as well as warming of blood and fluid transfusion, and monitoring of other body temperature protection measures. Before the second surgery, catheterization, drainage, and manometry of the cerebrospinal fluid were performed. There was no postoperative neurological complication. The 2 surgeries did not require infusion of too much allogeneic blood products and some was empirical usage. After surgery, the patient's coagulation function had recovered well, showing that this technology can achieve relatively good therapeutic outcomes. Conducting PRP before cardiopulmonary bypass (CPB) and transfusing autologous platelet-rich plasma (aPRP) after reversal of heparin could reduce postoperative blood loss, the requirements for blood products transfusion during cardiovascular surgery. A higher mean platelet count in aPRP may improve the final outcome.^[8]^

## Author contributions

**Data curation:** Yi Chang.

**Formal analysis:** Yi Chang.

**Project administration:** Ayong Tian.

**Writing – original draft:** Yi Chang.

**Writing – review & editing:** Rongwei Zhang, Ayong Tian.

Ayong Tian orcid: 0000-0002-0183-8556.

## References

[1] Zhou SF Estrera AL Loubser P Autologous platelet-rich plasma reduces transfusions during ascending aortic arch repair: a prospective, randomized, controlled trial. Ann Thorac Surg 2015;99:1282–90. 25661906 10.1016/j.athoracsur.2014.11.007

[2] Zhou SF Estrera AL Miller CR Analysis of autologous platelet-rich plasma during ascending and transverse aortic arch surgery. Ann Thorac Surg 2013;95:1525–30. 23245451 10.1016/j.athoracsur.2012.09.054

[3] Miller CR Porat EE Estrera AL Analysis of short-term multivariate competing risks data following thoracic and thoracoabdominal aortic repair. Eur J Cardiothorac Surg 2003;23:1023–7. discussion 1027. 12829082 10.1016/s1010-7940(03)00157-x

[4] Suzuki S Davis CR Miller CR Cardiac function predicts mortality following thoracoabdominal and descending thoracic aortic aneurysm repair. Eur J Cardiothorac Surg 2003;24:119–24. discussion 124. 12853055 10.1016/s1010-7940(03)00170-2

[5] Johansson PI Solbeck S Genet G Coagulopathy and hemostatic monitoring in cardiac surgery: an update. Scand Cardiovasc J 2012;46:194–202. 22375889 10.3109/14017431.2012.671487

[6] Van Poucke S Stevens K Wetzels R Early platelet recovery following cardiac surgery with cardiopulmonary bypass. Platelets 2016;27:751–7. 27164510 10.3109/09537104.2016.1173665

[7] Aoki K Sugimoto A Nagasawa A Optimization of thromboelastography-guided platelet transfusion in cardiovascular surgery. Gen Thorac Cardiovasc Surg 2012;60:411–6. 22566265 10.1007/s11748-012-0070-y

[8] Zhai Q Wang Y Yuan Z Effects of platelet-rich plasmapheresis during cardiovascular surgery: a meta-analysis of randomized controlled clinical trials. J Clin Anesth 2019;56:88–97. 30708148 10.1016/j.jclinane.2019.01.018

